# Construction of a competitive endogenous RNA network and identification of potential regulatory axes in hypertrophic cardiomyopathy

**DOI:** 10.3389/fcvm.2025.1552060

**Published:** 2026-01-12

**Authors:** Rui Gao, Meilin Liu, Haoyi Yang, Lingfeng Zha, Ni Xia

**Affiliations:** 1 Department of Cardiology, Union Hospital, Tongji Medical College, Huazhong University of Science and Technology, Wuhan, China; 2 Hubei Key Laboratory of Biological Targeted Therapy, Union Hospital, Tongji Medical College, Huazhong University of Science and Technology, Wuhan, China; 3 Hubei Provincial Engineering Research Center of Immunological Diagnosis and Therapy for Cardiovascular Diseases, Union Hospital, Tongji Medical College, Huazhong University of Science and Technology, Wuhan, China

**Keywords:** bioinformatics analysis, competitive endogenous RNA network, hypertrophic cardiomyopathy, immune infiltration, lncRNA, microRNA

## Abstract

**Background:**

Hypertrophic cardiomyopathy (HCM) is a complex and heterogeneous cardiovascular disease, the pathogenesis of which remains unclear. In this study, we aimed to explore potential biomarkers and competitive endogenous RNA (ceRNA) network in HCM using integrated bioinformatics analysis.

**Methods:**

Three mRNA expression datasets relevant to HCM, along with one long non-coding RNA (lncRNA) dataset, were retrieved from the Gene Expression Omnibus database. Differential expression analysis was conducted using the “limma” package. Hub genes were subsequently explored through an integrated bioinformatics approach, which included weighted gene co-expression network analysis (WGCNA), protein-protein interaction (PPI) network construction, and feature selection methods. The expression levels and diagnostic accuracy of the candidate hub genes were validated in GSE141910. A ceRNA regulatory network was constructed by predicting interactions using miRDB, miRWalk, DIANA-LncBase, and lncRNASNP2 databases. Finally, immune cell infiltration analysis was performed to elucidate the immune landscape in HCM.

**Results:**

We intersected genes from three sources: 642 differentially expressed genes (DEGs) from GSE36961, 1,612 DEGs from GSE160997, and 2,930 genes from key WGCNA modules, yielding 162 common genes. A PPI network of these genes revealed 78 nodes, from which three pivotal clusters were identified. Feature selection methods converged on three hub genes (CD163, FCER1G, and CYP2J2), each demonstrating high diagnostic value. A ceRNA network was constructed, revealing five potential regulatory axes: SNHG1/miR-543/CD163, MEG8/miR-543/CD163, ZFAS1/miR-2110/FCER1G, SNHG14/miR-5001-5p/FCER1G, and TTN-AS1/miR-6740-3p/CYP2J2. Immune infiltration analysis indicated notable dysregulation of multiple immune cells in HCM, and the identified hub genes showed significant correlations with key immune subsets, including macrophages, regulatory T cells, and activated dendritic cells.

**Conclusion:**

Through integrated analysis, three hub genes linked to immune function (CD163, FCER1G, and CYP2J2) were discerned, and a corresponding ceRNA network was delineated. These results contribute to a renewed understanding of the pathogenic mechanisms in HCM.

## 1 Introduction

Hypertrophic cardiomyopathy (HCM), a predominant cause of sudden cardiac death among young people (1), has a traditionally reported prevalence of approximately 1 in 500 (0.2%) in the general population (2). However, recent evidence incorporating genetic screening and advanced cardiac imaging suggests that the true prevalence, including clinical cases and genotype-positive individuals, may be as high as 1 in 200 (3). Characterized by myocardial hypertrophy, asymmetric hypertrophy of the ventricular septum, and ventricular narrowing, HCM can lead to arrhythmic sudden death, heart failure (HF), and atrial fibrillation (4). As a common inherited cardiovascular disease, tremendous advances have been made in elucidating its diverse genetic mutation sites in HCM. Genetic findings demonstrate a strong association between HCM pathogenesis and dominant mutations within genes that code for the sarcomere's thick/thin myofilament contractile components or the neighbouring Z-disc (5). Among those, myosin heavy chain 7 (MYH7) and myosin binding protein C3 (MYBPC3) are the two most common mutations, which encode β-myosin heavy chain and myosin binding protein C, respectively (6, 7). Nevertheless, the correlation between sarcomere gene mutations and clinical outcomes in HCM patients remains unpredictable, a phenomenon termed genetic and phenotypic heterogeneity (8). Apart from that, causative gene mutations remain unidentified in approximately 70% of HCM cases, and the regulatory mechanisms driving disease initiation and progression are not fully elucidated (9).

Recently, accumulating evidence has shown that long non-coding RNAs (lncRNAs) and microRNAs (miRNAs) have emerged as critical modulators of many biological processes such as proliferation, differentiation, apoptosis, and metabolism (10). MiRNAs are a novel class of small non-coding RNAs (ncRNAs), which are 22–25 nucleotides in length and negatively regulate gene expression by directly targeting mRNAs for degradation or post-transcriptional repression (11). Studies have demonstrated that miR-214 directly targets and suppresses sirtuin 3 (SIRT3), leading to mitochondrial dysfunction and thereby promoting the development of angiotensin II (Ang-II)-induced cardiac hypertrophy (12). Downregulated miR-451 also regulates cardiac hypertrophy and dysregulated autophagy by directly targeting tuberous sclerosis complex 1 (TSC1) (13). In addition to miRNAs, lncRNAs are known as important regulators of cardiac pathology, which are defined as RNAs with transcripts > 200 nucleotides in length with no protein-coding capacity (14). LncRNA Chast was reported to promote cardiac hypertrophy by repressing Pleckstrin homology domain-containing protein family M member 1 (PLEKHM1) and impairing cardiomyocyte autophagy (15). Consequently, ncRNAs might serve specific roles in the regulation of cardiovascular diseases, including HCM. Notably, lncRNAs contain miRNA response elements, having sponge-like effects on numerous miRNAs, acting as competitors of endogenous RNAs. Subsequently, lncRNAs thus regulate target mRNA degradation by interacting with miRNAs, which is called the competitive endogenous RNA (ceRNA) regulatory mechanism (16). Accumulating evidence has indicated that the ceRNA network is essential to understand the regulatory mechanisms in HCM (17).

Moreover, a previous study has appreciated that the early stage of HCM manifests injury-mediated inflammation and neutrophil extracellular traps, while a late phase was myocardial fibrosis, ventricular remodeling, and ultimately HF (18). Other studies have also shown that cardiac hypertrophy was accompanied by inflammatory signals as well as immune cell activation, which contributed to hypertrophic and fibrotic responses (19–21). Nevertheless, few studies have explored the relationship between lncRNA-related ceRNA networks and immune cell infiltration in HCM.

Taken together, we aimed to identify potential genes and elucidate the possible molecular basis of the lncRNA-miRNA-mRNA co-expression regulatory network in HCM, thereby advancing the understanding of its pathogenesis. In this study, original data encompassing HCM and control groups were sourced from the National Center for Biotechnology Information Gene Expression Omnibus (NCBI GEO) database. Then, we got differentially expressed genes (DEGs), followed by hub gene screening via weighted gene co-expression network analysis (WGCNA) and feature selection. Besides, we also sought to define the distinct immune infiltration profile associated with HCM. Finally, based on the shared-miRNA principle of ceRNA regulation, a tripartite ceRNA network was constructed. This work may offer novel insights into HCM mechanisms and inform future research directions.

## 2 Methods

### 2.1 Microarray data

Three HCM-related gene expression datasets (GSE36961, GSE160997, and GSE141910) and one ncRNA dataset (GSE68316) were downloaded from the NCBI GEO^1^ database. The dataset GSE36961, generated from GPL15839 (Illumina HumanHT-12 V3.0 expression bead chip) platform, consists of surgical myectomy tissues from 106 patients with HCM and 39 control donor cardiac tissues (22). Samples for GSE160997 were obtained from the anterior septal tissues of 18 patients with HCM and 5 healthy controls, which were generated on the GPL11154 Illumina HiSeq 2000 platform (23). The validation dataset GSE141910 was from the GPL16791 Illumina HiSeq 2500 platform, including left ventricular transcriptomes from a cohort comprised of patients with peripartum cardiomyopathy, HCM, dilated cardiomyopathy, and 166 non-failing healthy controls (24, 25). It was an original RNA sequencing dataset downloaded from the GEO database, and only 166 healthy controls and 28 patients with HCM were selected for bioinformatics analysis in this study. GSE68316 came from GPL20113 (CapitalBio Human LncRNA Microarray v2.0) and contained myocardial tissues from 7 patients with HCM as well as 5 healthy controls, and only lncRNA expression profiles were included for further analysis (26).

### 2.2 Data preprocessing and differential expression analysis

R^2^ software (version 4.1.3) was used to analyze the obtained datasets. For GSE36961, the standardized probe expression matrix was downloaded, which had already been annotated, so it was directly used in the next step of differential expression analysis. In addition, the other three raw datasets were preprocessed using the “limma” package. Then we chose the BioMart^3^ database to transform the Ensemble IDs in these datasets into respective gene symbols. Probes without corresponding gene symbols were eliminated, while the average value was calculated as the expression of the gene. The R package “limma” was also applied to identify DEGs between the control and HCM groups with criteria of |logFC (fold change) | > 0.58 (approximately corresponding to a 1.5-fold change) and adjusted *p*-value (adj. p) <0.05, and |logFC| > 1 and adj. *p* < 0.05 were used as the screening standard for differentially expressed lncRNAs (DElncRNAs) in GSE68316. All gene symbols used in this study are in accordance with the approved nomenclature from the Human Gene Nomenclature Committee.

### 2.3 Functional and pathway enrichment analysis

To explore the principal biological functions and pathways involved in HCM, enrichment analyses were conducted. First, Gene Ontology (GO) analysis and Kyoto Encyclopedia of Genes and Genomes (KEGG) pathway analysis were performed using the DAVID database. GO enrichment covers three categories: biological processes (BPs), cellular components (CCs), and molecular functions (MFs). Terms with a false discovery rate (FDR) <0.05 were deemed statistically significant. Subsequently, immunologic signature gene sets (c7.immunesigdb.v2023.1.Hs.symbols.gmt) from the Molecular Signatures Database (MsigDB)^4^ were utilized as the reference for gene set enrichment analysis (GSEA) (27), where gene sets meeting the thresholds of adj. *p* < 0.05 and FDR *q*-value < 0.05 were considered significantly enriched.

### 2.4 Weighted gene co-expression network analysis

To identify clinically key modules of HCM, using the “WGANA” package, genes with the cut-off point of adj. *p* < 0.001 from GSE36961 were imported for WGCNA to construct the gene co-expression network. First, the correlation network was constructed with an appropriate soft threshold power *β* = 7, realizing a scale-free topology criterion of R^2^ > 0.80. Then, the average linkage hierarchical clustering method was applied to cluster genes into different modules with different colors. Modules were merged using a threshold of 0.15, with each retained module required to contain a minimum of 30 genes. Third, hierarchical clustering was applied to delineate gene modules characterized by high intra-modular connectivity. The Spearman coefficient was calculated to assess the correlation between each module and HCM phenotype. Finally, only modules that met the following criteria were regarded as pivotal modules: (1) their correlation coefficient with HCM was >0.70 while *p* < 0.05; (2) the correlation coefficient between gene significance and module memberships for HCM was also >0.70. Genes residing in modules meeting these criteria were extracted for subsequent analysis.

### 2.5 Protein-protein interaction (PPI) network construction

The STRING^5^ database (version 11.5), an online tool to excavate functional protein associations (28), was used to construct a PPI network with a high-confidence set (score >0.70). This obtained PPI network was further visualized using Cytoscape^6^ software (version 3.9.1). Subsequently, the Molecular Complex Detection (MCODE) plugin was employed to select clusters with the highest degree of connectivity, genes in which were considered as candidates.

### 2.6 Feature selection

To minimize the risk of bias in screening significant variables, three different feature selection methods, the least absolute shrinkage and selection operator (LASSO), random forest (RF), and the support vector machine recursive feature elimination (SVM-RFE), were applied to further filter candidate genes for HCM. LASSO regression utilizes regularization for variable selection in high-dimensional data (29). It was implemented using the “glmnet” R package with a 5-fold cross-validation to determine the optimal penalty parameter. The minimum lambda (lambda. min) was selected as a suitable lambda value to identify the variables in the models. The RF classifier, an ensemble of decision trees, was constructed via the “randomForest” R package (30). Its performance was evaluated using 10-fold cross-validation, and genes with a relative importance score >2 were retained. SVM-RFE, a supervised algorithm for classification, was executed with the “e1071” R package, also employing 10-fold cross-validation (31). The intersections of genes from the above three methods were considered hub genes in HCM.

### 2.7 Receiver operating characteristic (ROC) curve building

The diagnostic performance of each hub gene was evaluated using ROC analysis, implemented with the R package “pROC” for visualization. The area under the curve (AUC) and 95% confidence interval (CI) of hub genes were calculated in validation datasets GSE141910, while an AUC > 0.800 was considered the ideal diagnostic value.

### 2.8 LncRNA-miRNA-mRNA network building

Following systematic bioinformatics analyses and feature selection, candidate hub genes were designated for ceRNA network construction. First, potential miRNAs targeting these hub genes were predicted using the combined resources of the miRDB^7^ database (32) and the miRWalk^8^ tool (33) to ensure the robustness of miRNA-mRNA interactions. Subsequently, lncRNA-miRNA interactions were predicted by querying the DIANA-LncBase v2^9^ database (34) and lncRNASNP2^10^ database (35). Next, we selected the intersection between predicted lncRNAs and DElncRNAs in GSE68316 to construct the lncRNA-miRNA-mRNA regulatory network and removed the negatively related mRNA-lncRNA co-expression interactions. Finally, the resultant ceRNA network was visualized through the Sankey diagram.

### 2.9 Single-sample gene set enrichment analysis (ssGSEA)

Given the functional enrichment results, which primarily linked the screened genes to immune-related processes, we proceeded to characterize the immune infiltration landscape in HCM using dataset GSE36961. This was achieved by applying the ssGSEA algorithm (“GSVA” package) (36), which quantifies cell-type-specific abundance using a published signature matrix of 782 marker genes for 28 immune cell types (37). Then we analyzed differential expression in immune cells infiltration between the HCM and control groups, which was visualized by bar plots. Besides, correlations between HCM-related hub genes and these immune cells were assessed using Spearman correlation analysis, followed by visualization using the “ggplot2” package.

### 2.10 Statistical analysis

All analyses were conducted in R (version 4.1.3). The PPI network was built and analyzed with a confidence threshold set of score >0.70. Diagnostic biomarker efficacy was evaluated by generating ROC curves via the “pROC” package, and the corresponding AUC values were calculated as well. The relationships between hub genes and infiltrating immune cells were examined using Spearman correlation analysis. The negatively related mRNA-lncRNA co-expression interactions and positively related mRNA-miRNA and lncRNA-miRNA were selected to construct the ceRNA network. Statistical significance was defined as a two-sided *p*-value < 0.05.

## 3 Results

### 3.1 Identification of DEGs and DElncRNAs in HCM

**Figure 1:**
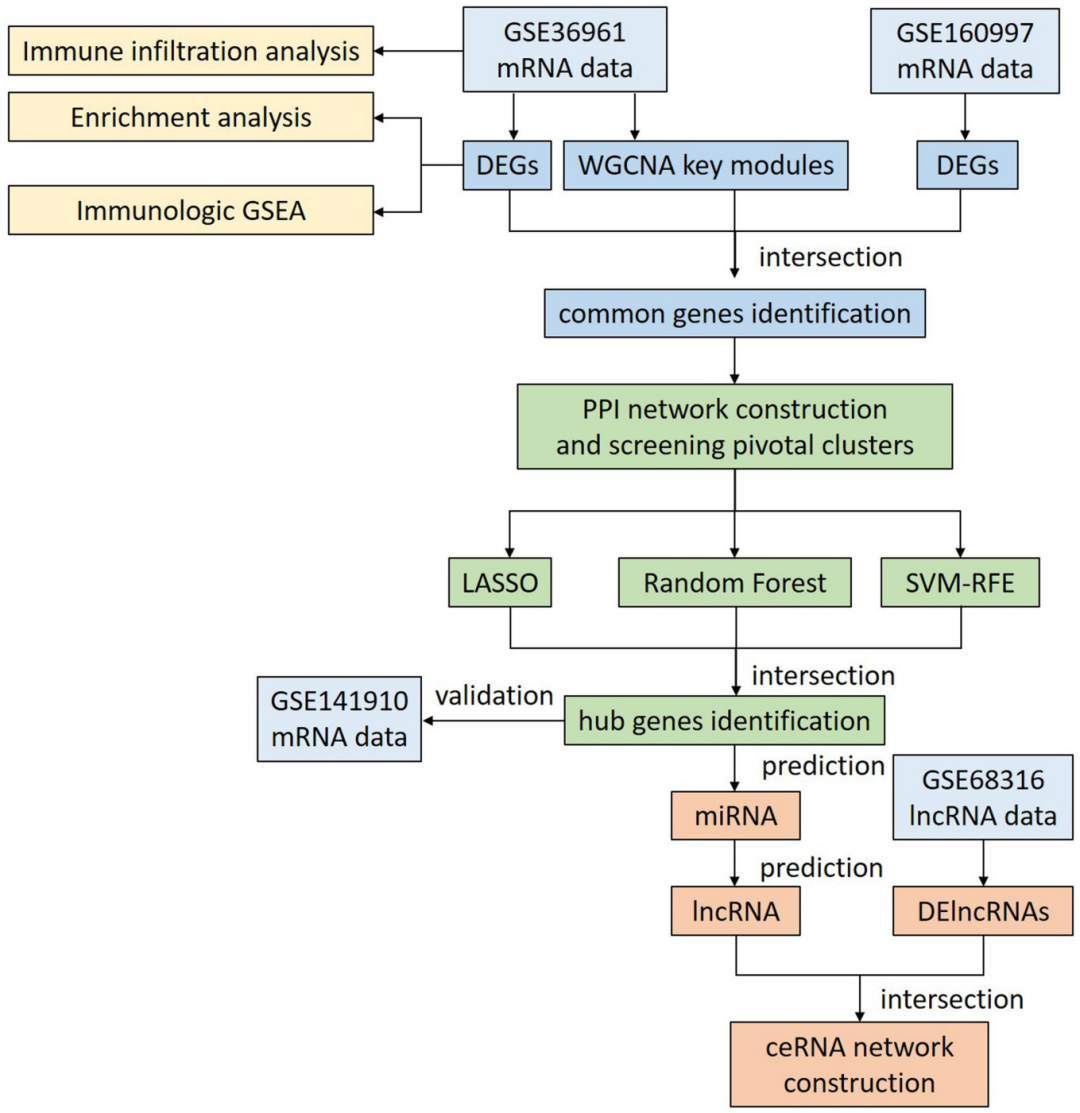
Flowchart of the overall analysis. DEGs, differentially expressed genes; DElncRNAs, differentially expressed lncRNAs; GSEA, gene set enrichment analysis; WGCNA, weighted gene co-expression network analysis; PPI, protein-protein interaction; LASSO, least absolute shrinkage and selection operator; SVM-RFE, support vector machine recursive feature elimination; ceRNA, competitive endogenous RNA.

**Figure 2:**
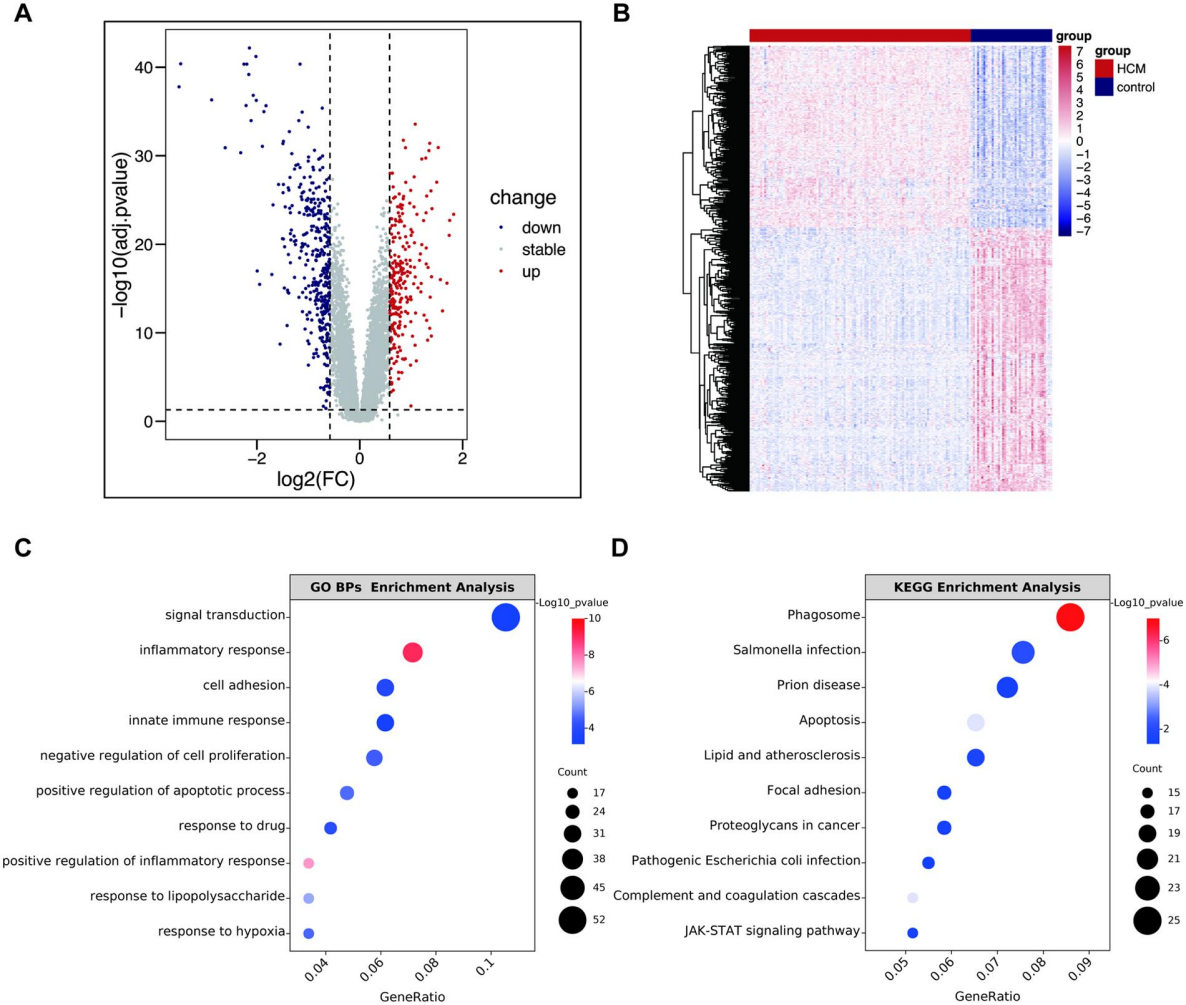
Identification of DEGs in GSE36961 and enrichment analysis. **(A)** The volcano plot of GSE36961. **(B)** Heatmap of DEGs in GSE36961. **(C)** The top 10 significant terms of GO BPs enrichment analysis. **(D)** The top 10 significant terms of KEGG enrichment analysis. GO, Gene Ontology; KEGG, Kyoto Encyclopedia of Genes and Genomes; BPs, biological process; FC, fold change; HCM, hypertrophic cardiomyopathy; other abbreviations are shown in Figure 1.

The study design is presented in the flowchart shown in Figure 1. To systematically identify transcriptional alterations in HCM, we first performed differential expression analysis on the largest dataset, GSE36961. We identified a total of 642 DEGs (262 upregulated and 380 downregulated), indicating widespread transcriptional dysregulation in HCM myocardial tissue (Figures 2A,B). In another HCM dataset, GSE160997, we found that 824 DEGs were significantly upregulated, and 788 DEGs were downregulated. Regarding the lncRNA dataset, 1,425 DElncRNAs were screened out (965 upregulated and 460 downregulated). This preliminary finding underscores the potential significant role of ncRNAs in HCM and provides a foundation for subsequent ceRNA network construction.

### 3.2 Functional and pathway enrichment analysis of DEGs from GSE36961

To elucidate the potential biological functions of these DEGs, we performed GO and KEGG pathway enrichment analyses. As depicted in Figure 2C, the GO BPs analysis revealed significant enrichment of DEGs in terms such as “signal transduction”, “inflammatory response”, and “innate immune response”, whereas the results of CCs and MFs analysis were listed in Supplementary Figure S1. Concurrently, KEGG pathway analysis indicated enrichment in “Phagosome”, “Lipid and atherosclerosis”, “Complement and coagulation cascades”, and “JAK/STAT signaling pathway”, indicating these pathways may critically contribute to HCM pathogenesis (Figure 2D). This finding is pivotal as it directly links our transcriptomic data to the increasingly recognized immune and inflammatory hypotheses in HCM, providing a strong rationale for our subsequent immune infiltration analysis.

### 3.3 WGCNA and pivotal modules identification

**Figure 3:**
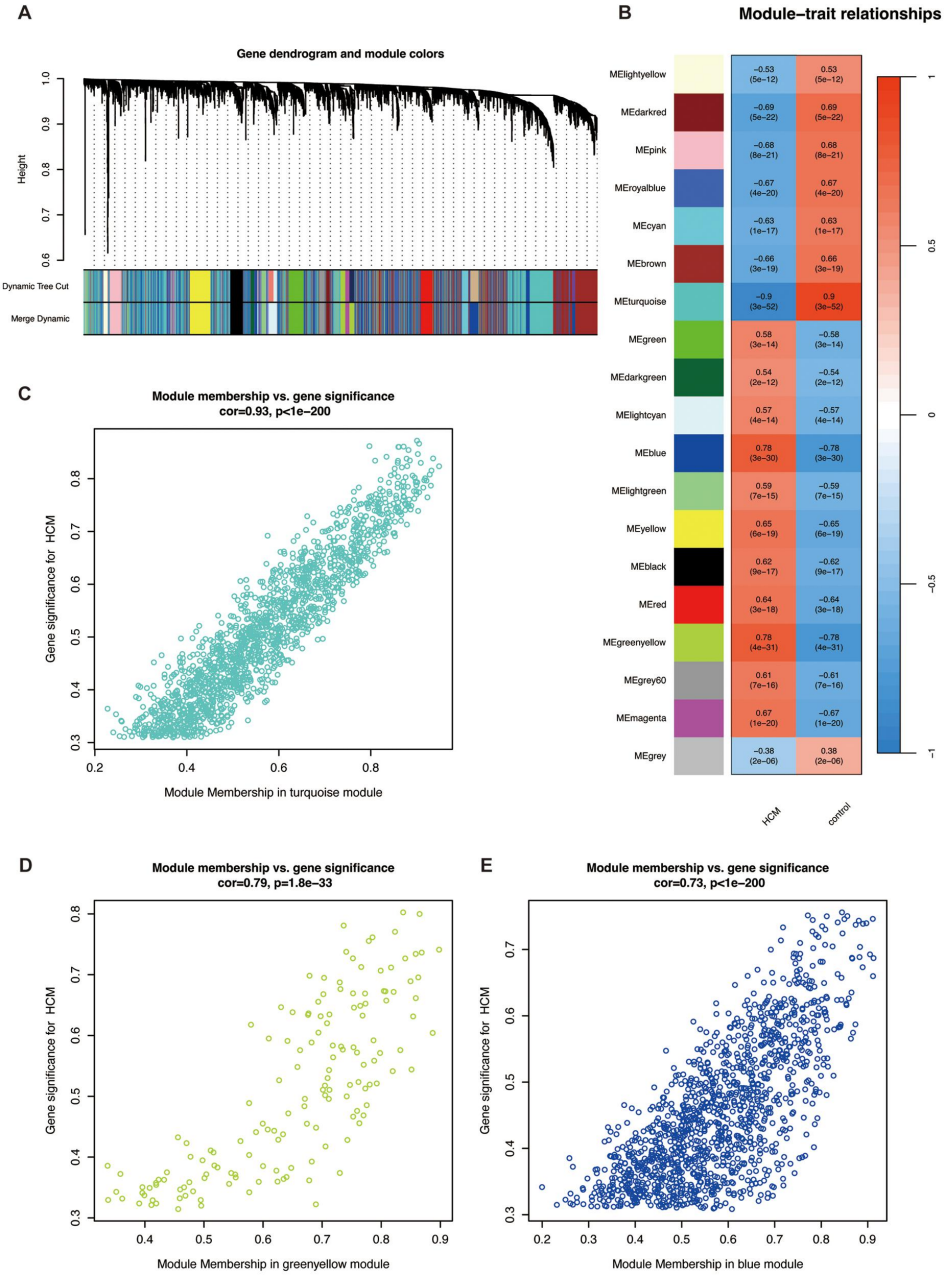
Weighted gene correlation network analysis. **(A)** Cluster dendrogram of modules. **(B)** Heatmap of module-HCM relationships. Blue represents a negative correlation, and red represents a positive correlation. Correlation plot between module membership and gene significance of genes included in the **(C)** turquoise, **(D)** greenyellow, and **(E)** blue modules. Abbreviations are shown in Figures 1, 2.

Genes with adj. *p* < 0.001 in GSE36961 were selected for WGCNA, and a scale-free co-expression network was established. We chose the soft threshold power *β* = 7 with scale-free R^2^ > 0.80 based on the scale independence and mean connectivity (Figure 3A). According to the threshold for module merging, these genes were clustered into 19 co-expression modules through the average linkage hierarchical clustering method. Different modules were represented by different colors, including black, blue, brown, cyan, darkgreen, darkred, green, greenyellow, grey, grey60, lightcyan, lightgreen, lightyellow, magenta, pink, red, royalblue, turquoise, and yellow (Figure 3B). Genes in the gray module, indicative of non-co-expressed genes, were excluded from subsequent analysis. We then computed the correlation between module membership and gene significance for the HCM phenotype. According to the above selection, 2,930 HCM-related genes in the turquoise, greenyellow, and blue modules were identified (Figures 3C–E). This suggests that genes within these modules likely operate as functional units, collectively driving HCM pathogenesis. Therefore, we defined these as key modules for downstream analysis.

### 3.4 Common genes identification and PPI network construction

**Figure 4:**
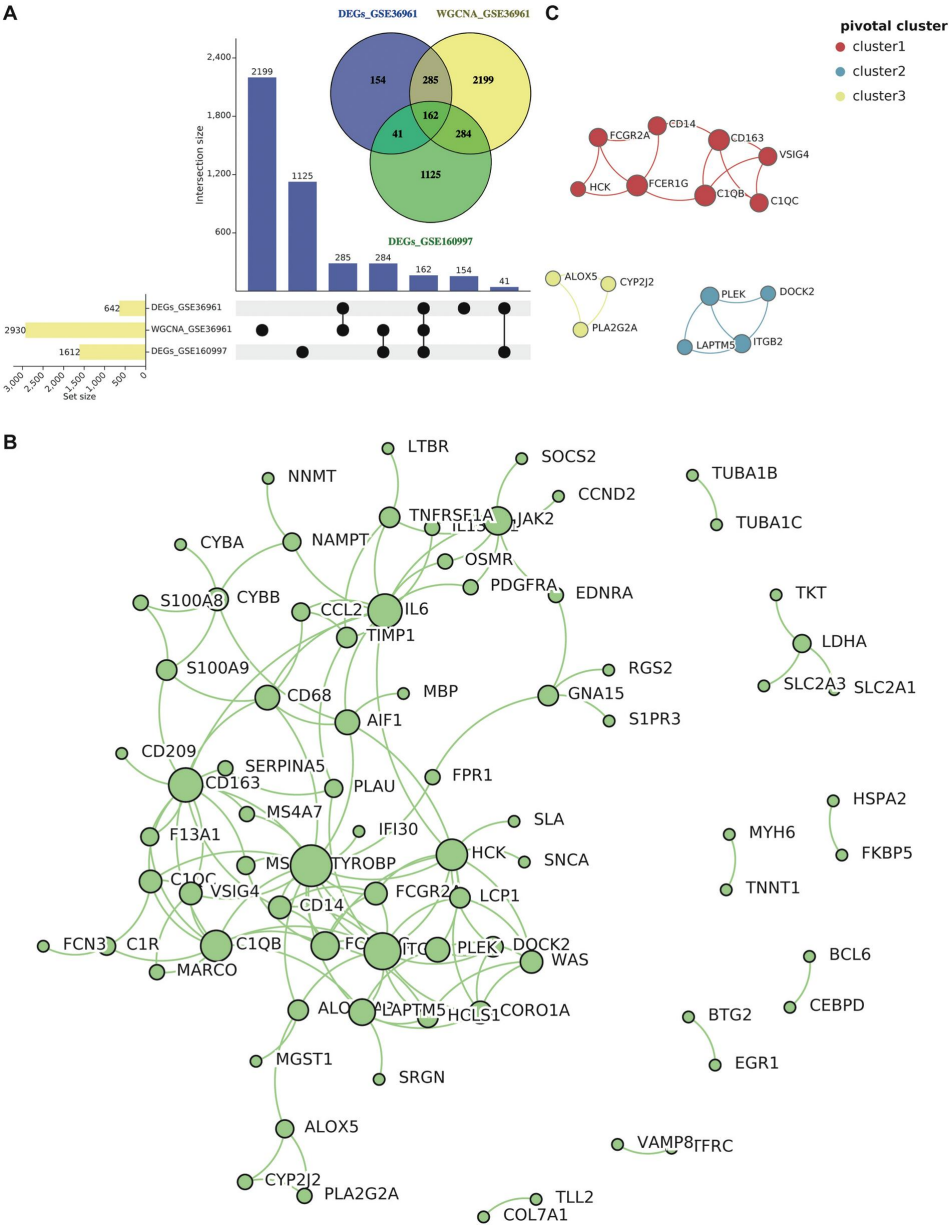
Common gene selection and PPI network construction. **(A)** The intersection of DEGs in GSE36961 and GSE160997, as well as WGCNA key modules. **(B)** The PPI network of the common genes (score > 0.70) contained 78 nodes and 131 interaction pairs. **(C)** The three most pivotal clusters include 15 correlative genes. Abbreviations are shown in Figures 1–3.

We integrated genes identified from differential expression analyses (GSE36961 and GSE160997) with those from the WGCNA key modules. The intersection yielded 162 common genes (Figure 4A). These genes simultaneously satisfy the stringent dual criteria of being both differentially expressed and co-expressed, marking them as high-confidence core regulatory factors in HCM. Based on the 162-gene set, a PPI network was generated to determine genes that directly interact with each other. After hiding the disconnected nodes and setting the minimum required interaction score as high-confidence (score > 0.70), 78 protein-coding nodes and 131 edges were identified in the PPI network. Then this network was visualized via Cytoscape (Figure 4B), and we obtained the most pivotal clusters through the MCODE plug-in. As presented in Figure 4C, 15 correlative genes were picked out from three pivotal clusters.

### 3.5 Hub gene selection via feature selection

**Figure 5:**
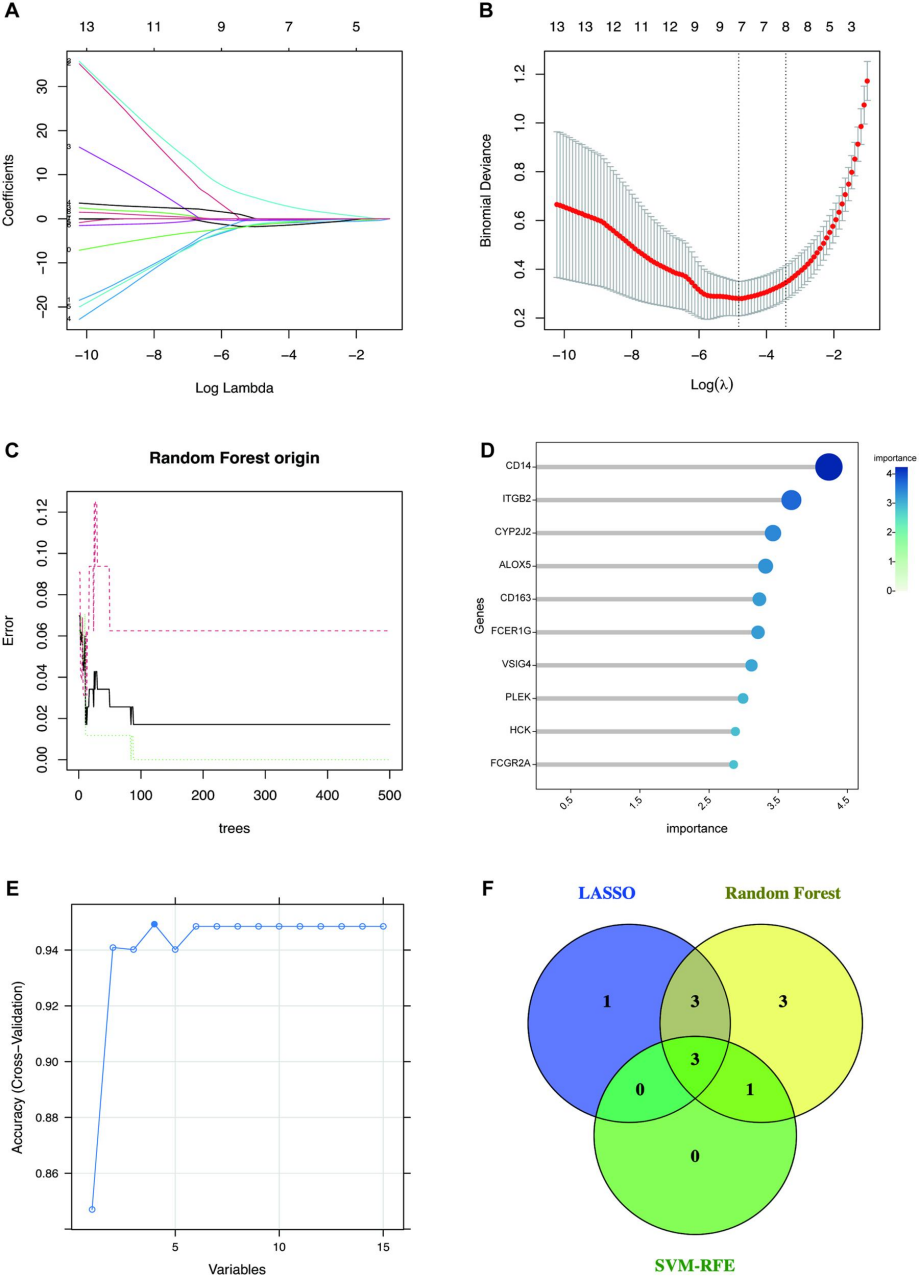
Feature selection methods for identifying candidate hub genes. **(A,B)** Biomarker screening in the LASSO model. The number of genes (*n* = 7) corresponding to the lowest point of the curve is the most suitable for HCM diagnosis. **(C)** The RF algorithm shows the error in HCM. **(D)** The top 10 genes are ranked with an importance score using the RF algorithm. **(E)** SVM-RFE algorithm for feature selection, variables = 4 corresponding to the highest accuracy. **(F)** The Venn diagram depicted the intersection of genes shared by three distinct methods. SVM-RFE, support vector machine-recursive feature elimination; LASSO, the least absolute shrinkage and selection operator; RF, random forest. Abbreviations are shown in Figures 1–4.

To identify the most robust and diagnostically promising hub genes from the candidate pool, we employed three complementary feature selection methods—LASSO, RF, and SVM-RFE—to mitigate bias inherent to any single method. Figures 5A,B indicate that the LASSO regression algorithm selected 7 feature variables at the minimum deviance. The RF algorithm ranked genes by variable importance score, with the top 10 most significant genes selected (Figures 5C,D). When the accuracy is highest, exactly as in Figure 5E, the SVM-RFE algorithm filtered 4 candidate genes. The Venn diagram showed that the intersection of candidate genes identified by these three feature selection methods was three hub genes, including CD163, FCER1G, and CYP2J2 (Figure 5F). These three genes possess the most powerful and consistent discriminative power for distinguishing HCM from control samples.

### 3.6 Validation of the expression and diagnostic value evaluation of hub genes in GSE141910

**Figure 6:**
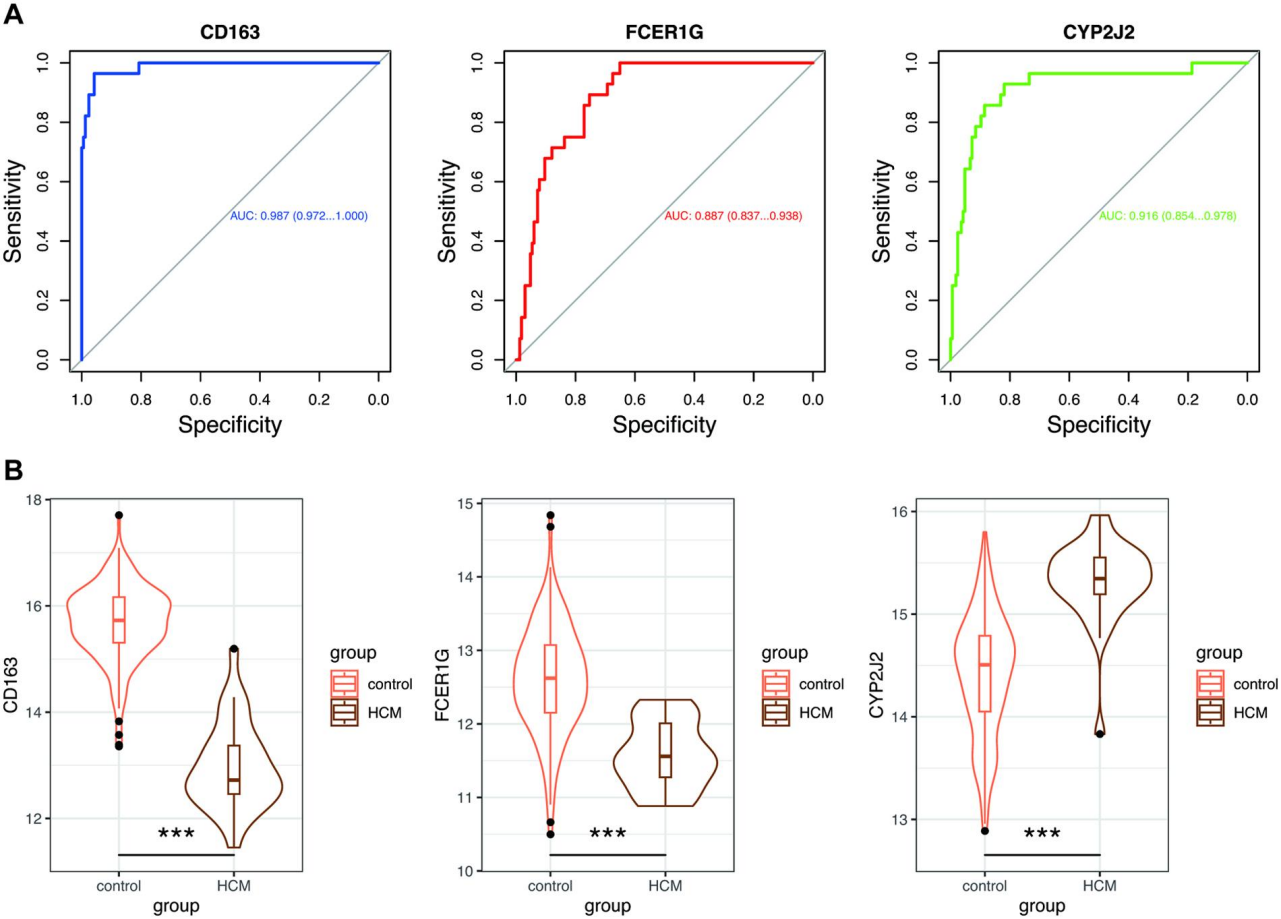
Validation of hub genes in GSE141910. **(A)** ROC curves estimated the diagnostic performance of three hub genes. **(B)** Violin plots showed the expression of hub genes between the HCM and control groups. Wilcoxon test: *, *p* < 0.05; **, *p* < 0.01; ***, *p* < 0.001. ROC, receiver operating characteristic; AUC, area under the curve; other abbreviations are shown in Figures 1–5.

To validate the expression profiles and diagnostic potential of hub genes, we drew ROC curves and analyzed gene expression between HCM patients and control subjects using the independent dataset GSE141910. The AUC and 95% CI for each gene from the HCM validation dataset are shown in Figure 6A: CD163 (AUC: 0.987, CI 0.972–1.000), FCER1G (AUC: 0.887, CI 0.873–0.938), and CYP2J2 (AUC: 0.916, CI 0.854–0.978), which demonstrated optimal diagnostic value with excellent specificity and sensitivity. Furthermore, as shown in Figure 6B, the three hub genes were significantly differentially expressed between the HCM and control groups.

### 3.7 MiRNAs prediction and ceRNA network construction

**Figure 7:**
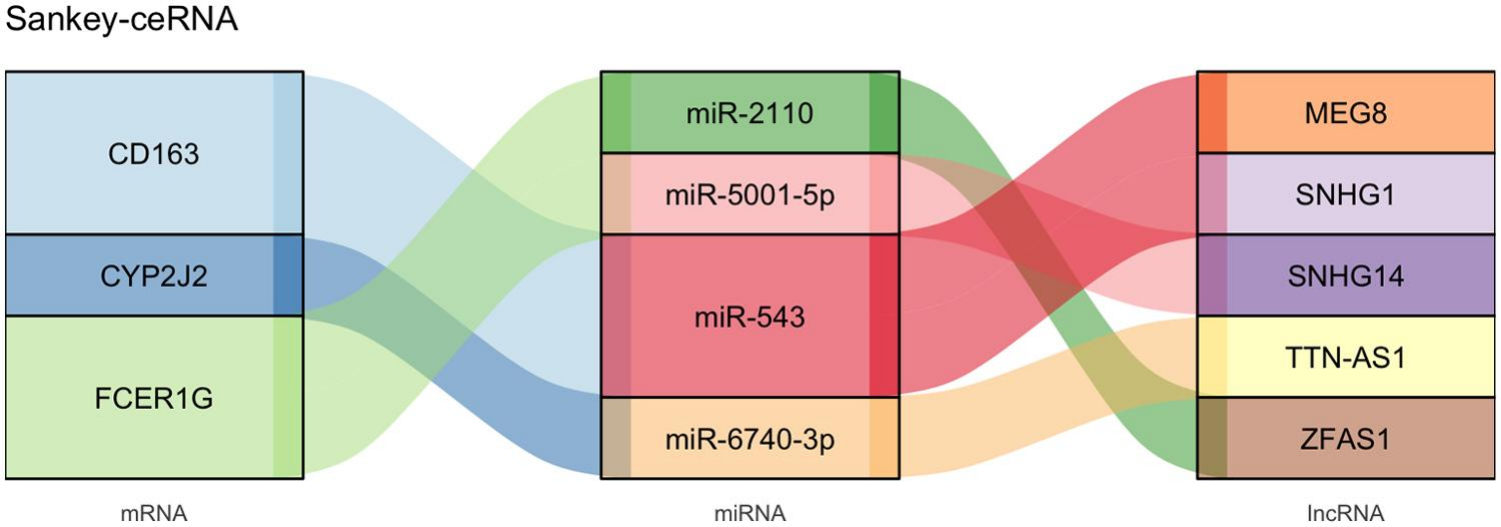
Sankey diagram of the final lncRNA-miRNA-mRNA network. MiRNAs targeting hub genes were predicted via the miRDB with the miRWalk database, and lncRNA-miRNA interactions were predicted by the DIANA and the lncRNASNP2 database. The ceRNA network was constructed by Cytoscape and visualized through the Sankey diagram. ceRNA, competitive endogenous RNA; lncRNA, long non-coding RNA; miRNA, microRNAs.

Guided by the ceRNA hypothesis, we proceeded to construct a lncRNA-miRNA-mRNA regulatory network centered on the validated hub genes. Two databases mentioned above were intersected to predict miRNAs putatively targeting mRNA, and a total of 30 miRNAs were identified, respectively, including 12, 15, and 3 miRNAs corresponding to CD163, FCRE1G, and CYP2J2. Then we predicted the potential interactions between these miRNAs and lncRNAs through the DIANA and lncRNASNP2 database, and there are 9 miRNAs with existing lncRNA-miRNA interactions. Among these miRNAs, miR-4421, miR-543, and miR-5699-3p regulate CD163; miR-2110, miR-4425, and miR-5001-5p regulate FCER1G; miR-1255b-2-3p, miR-6740-3p, and miR-6780a-3p regulate CYP2J2. After excluding pairs with negative mRNA-lncRNA co-expression correlation, we intersected the lncRNAs predicted to bind these miRNAs with the DElncRNAs in GSE68316. This process identified 5 key lncRNAs, including MEG8, SNHG1, ZFAS1, SNHG14, and TTN-AS1. The resulting ceRNA regulatory network was then presented as a Sankey diagram in Figure 7.

### 3.8 ssGSEA and immune cell infiltration analysis

**Figure 8:**
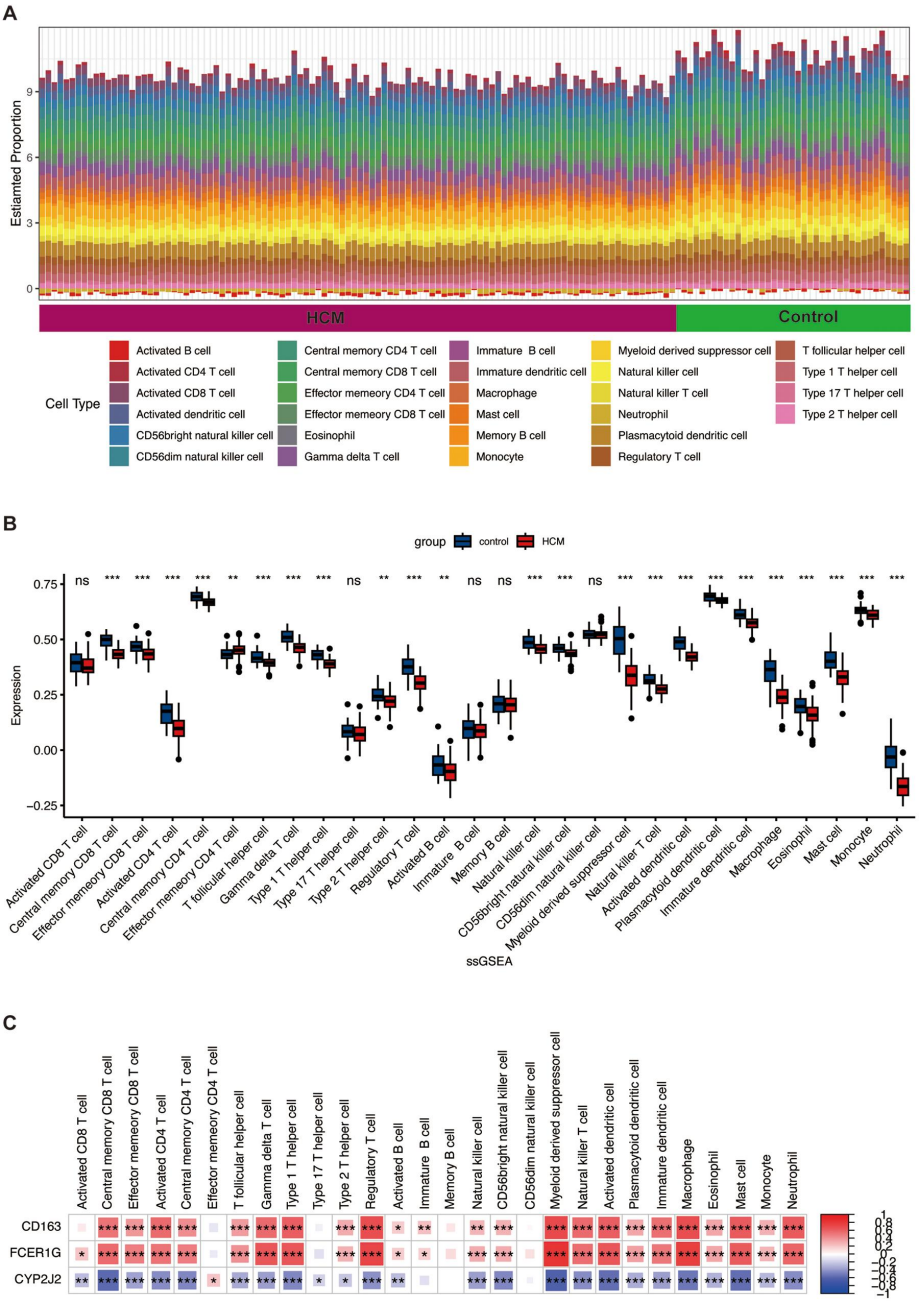
Immune cell infiltration analysis between HCM and control groups. **(A)** The proportion of 28 kinds of immune cells in different samples is visualized by the barplot. **(B)** The comparison regarding the proportion of 28 kinds of immune cells between the HCM and control groups is visualized by the barplot. **(C)** Correlation analysis between expression levels of hub genes and immune cells. Red represents a positive correlation, and blue represents a negative correlation. *, *p* < 0.05; **, *p* < 0.01; ***, *p* < 0.001. ssGSEA, single-sample gene set enrichment analysis; other abbreviations are shown in Figures 1–7.

Since the identified DEGs in GSE36961 were mainly enriched in immune and inflammation response, as well as the complement and coagulation cascades, we excavated immune cell infiltration based on GSE36961, following the use of the ssGSEA algorithm. Compared to controls, HCM samples showed significant alterations in the proportions of various immune cells. Notably, the infiltration of activated CD4^+^ T cells, regulatory T cells (Tregs), macrophages, and activated dendritic cells (DCs), among others, was significantly lower in the HCM group (Figures 8A,B).

### 3.9 Correlation analysis between hub genes and infiltrating immune cells

Associations among the three hub genes (CD163, FCER1G, CYP2J2) and infiltrating immune cells were assessed using Spearman correlation analysis in the context of HCM. The results indicated the presence of significant correlations between the expression levels of these genes and specific subsets of the dysregulated immune cells (Figure 8C). For instance, CD163 and FCER1G expression showed positive correlations with macrophages and Tregs, whereas CYP2J2 showed negative correlations. These strong correlations directly link our hub genes to the immune landscape of HCM, implying they may function as potential bridges connecting immune cell infiltration with cardiomyocyte pathology.

### 3.10 Enrichment analysis of GSEA immune signature gene sets

**Figure 9:**
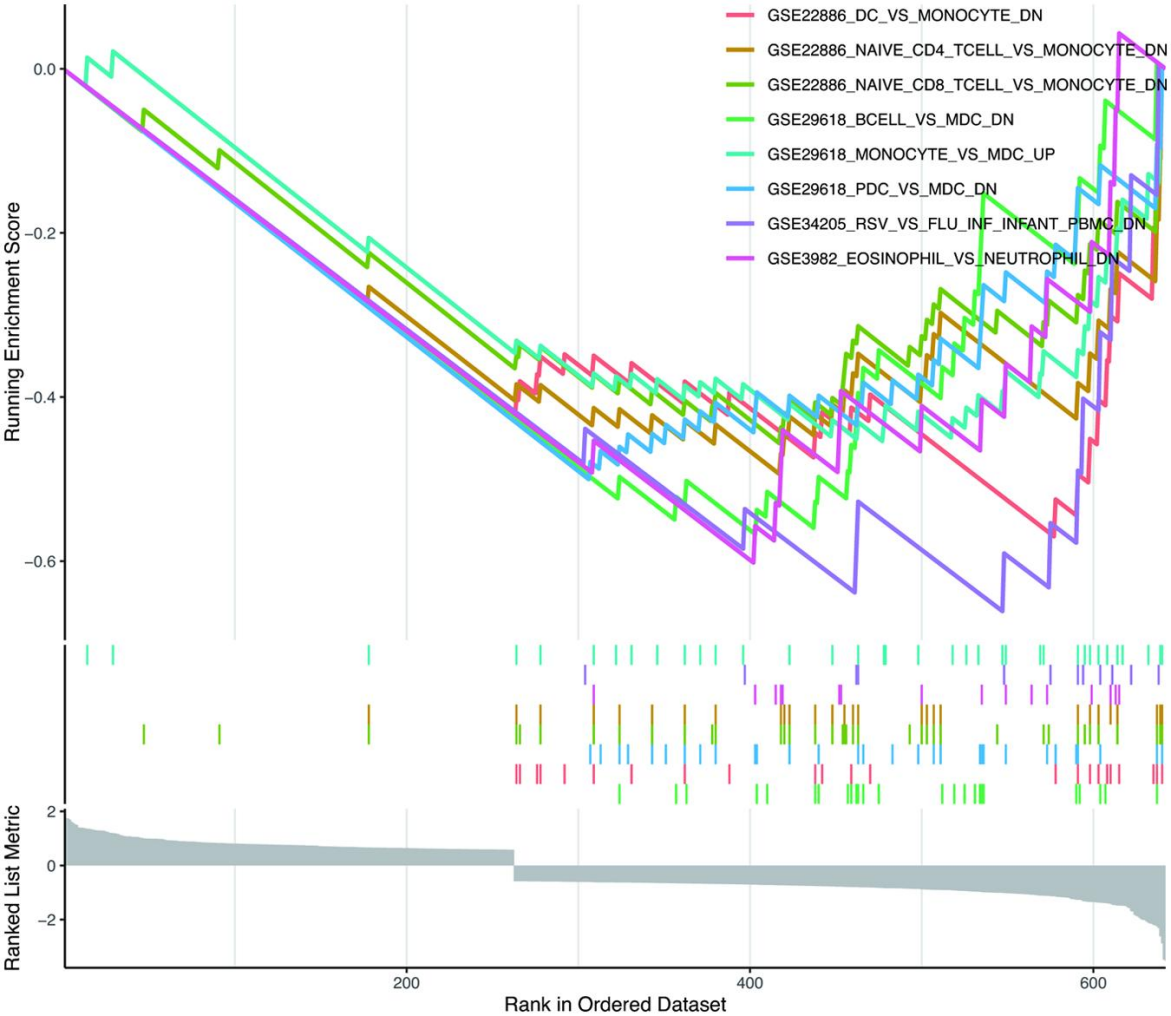
Enrichment plot for GSEA based on DEGs in GSE36961. The immunologic signature gene sets (c7.immunesigdb.v2023.1.Hs.symbols.gmt) were downloaded from the Molecular Signatures Database as the reference, where gene sets with adj. *p* < 0.05 and FDR *q*-value < 0.05 were considered significantly enriched. GSEA, gene set enrichment analysis; FDR, false discovery rate; other abbreviations are shown in Figures 1–8.

To probe the immune mechanisms underlying HCM progression, we performed GSEA on the DEGs from GSE36961, using immunologic signature gene sets from the MsigDB as the reference. Applying stringent criteria (|normalized enriched score| > 2, FDR *q*-value < 0.05, adj. *p* < 0.05), a total of 60 gene sets were identified as significantly enriched. These enriched sets were primarily associated with immune cell lineages, including monocytes, T cells, B cells, neutrophils, and DCs (Figure 9). This analysis echoes and extends our immune infiltration results, collectively building a multi-faceted evidence chain for immune dysregulation in HCM.

## 4 Discussion

HCM is a significant hereditary cardiomyopathy and a major etiology of sudden cardiac death (1). Due to the marked diversity and heterogeneity in the associated molecular pathways, the exact pathogenesis of HCM remains undetermined. Recently, studies have shown that ncRNAs have been identified as novel regulatory factors in HCM. The lncRNA ADAMTS9-AS1, found downregulated in HCM patient serum, was shown to inhibit hypertrophy via the miR-185-5p/lysine acetyltransferase 7 (KAT7) pathway (38). The lncRNA CYTOR exerts a protective effect against cardiac hypertrophy by serving as a molecular sponge for miR-155, thereby upregulating IKKi and modulating the NF-*κ*B signaling pathway (39). However, the mechanisms of these ncRNAs involved in the pathogenesis of HCM require much elaboration, and the lncRNA-miRNA-mRNA network may play an important role. Thus, we have made a systematic analysis of HCM transcriptional data, aiming to explore and identify relevant potential biomarkers and construct a ceRNA network, thereby contributing to a more integrated understanding of its molecular and pathological basis.

This analysis was initiated with the acquisition and processing of the GSE36961 dataset from the GEO database. DEGs between patients with HCM and healthy controls were identified, and GO and KEGG enrichment analyses were performed. The results indicated that these genes were mainly enriched in inflammation and immune response, suggesting the potential role that immune cells may play in the occurrence and progression of HCM. Further, to search for hub genes of HCM, we clustered genes of GSE36961 into different modules and calculated the correlations between modules and HCM through WGCNA. The turquoise, greenyellow, and blue modules were identified as the key modules with high correlation coefficients, and genes in these modules were identified as candidate genes. Another HCM dataset, GSE160997, was also obtained from the GEO database, and DEGs were confirmed using the same screening criteria as GSE36961. The intersection of these gene sets yielded 162 common genes. Next, we constructed a PPI network and excavated three pivotal clusters. Based on genes in the above clusters, three feature selection methods were used to screen out hub genes. Finally, according to the mRNA-miRNA and miRNA-lncRNA interactions predicted by the online databases, a ceRNA network was constructed, involving three mRNAs, four miRNAs, and five lncRNAs, which may offer valuable insights for diagnostic and therapeutic strategies for HCM.

Recent studies have established that immune cells are key regulators of both cardiac homeostasis and disease, actively contributing to the pathogenesis of disorders such as HCM (40, 41). Furthermore, single-cell transcriptomic analysis demonstrated that immune cell-mediated communication with other cardiac cell types is a critical mechanism underlying pathological cardiac hypertrophy (42). As a result, to explore the potential mechanisms of immune function during HCM progression, immune cell infiltration analysis and immunologic GSEA were performed on GSE36961. It turned out that the course of HCM was associated with the abnormal regulation of T cells, B cells, macrophages, monocytes, DCs, etc, which might elucidate that the involvement and regulation of inflammation and immune response were significant in the pathogenesis of HCM to an extent.

We first proposed that CD163 expression was regulated via the SNHG1/miR-543/CD163 and MEG8/miR-543/CD163 axis. As a surface molecule, CD163 encodes a membrane protein consisting of the scavenger receptor cysteine-rich domain and is primarily and highly expressed on the surface of monocytes and macrophages (43). Monocytes and macrophages are well-established as pivotal regulators of the inflammatory and reparative responses in the heart, orchestrating key phases of tissue healing and remodeling following myocardial injury (44, 45). Previous research indicated that macrophages participate in the cardiac remodeling of HCM by modulating cardiomyocyte hypertrophy and fibroblast activation (46). Particularly, there are kinds of differentiated macrophage subtypes. Classically activated (M1) macrophages are typically pro-inflammatory cells active in initial inflammatory responses, which exhibit high levels of pro-inflammatory and phagocytic activities, whereas alternatively activated (M2) macrophages have exerted anti-inflammatory effects and take part in extinguishing inflammatory responses (47–49). As a recognized marker of M2 macrophages, CD163 is implicated in facilitating the phenotypic shift from M1 to M2, promoting the release of anti-inflammatory factors to help resolve inflammatory processes (50). Recent evidence indicates that CD163 deficiency aggravates left ventricular systolic dysfunction following pressure overload. This detrimental effect is mediated through the accumulation of damaged mitochondria in cardiomyocytes, highlighting the cardioprotective function of CD163^+^ macrophages (51). This finding aligns with our results. Immune infiltration analysis revealed a strong correlation between CD163 expression and monocyte/macrophage abundance in HCM, implying that CD163 may serve as an immune-associated biomarker in this condition. Notably, we observed downregulation of CD163 in HCM, which echoes previous reports (52) and further supports the hypothesis that a reduction in CD163^+^ macrophages may contribute to HCM pathogenesis.

In the previous study, lncRNA MEG8 is implicated in carcinogenesis (53, 54). Beyond its other roles, MEG8 has also been found to be intimately linked to the development and progression of inflammation. Xie et al. reported that MEG8 could suppress apoptosis and inflammatory responses in chondrocytes via downregulation of the PI3K/AKT signaling pathway (55). MEG8 was also proven to suppress the JAK2/STAT3 pathway in rat monocyte-derived macrophages, while the activation of the JAK2/STAT3 pathway could lead to cardiac fibroblast-to-myofibroblast conversion (56, 57). Presumably, the downregulation of MEG8 in HCM may promote cardiac fibrosis and further aggravate myocardial hypertrophy. LncRNA SNHG1 is aberrantly expressed in a wide range of human cancers, where it could function as a modulator of M2 macrophage polarization (58). Besides, recent studies have extended its functional relevance to cardiovascular diseases. Yan et al. found for the first time that SNHG1 could attenuate cardiomyocyte hypertrophy by targeting miR-15a, in other words, played a protective role against pathological cardiac hypertrophy (59). Collectively, the downregulation of SNHG1 in HCM is likely involved in aggravating cardiomyocyte hypertrophy.

The above analysis suggests that MEG8 and SNHG1 likely function as post-transcriptional regulators by sponging miR-543, a molecule documented to play a significant role in inflammatory processes. Supporting evidence shows that miR-543 is upregulated in viral myocarditis, where its inhibition alleviates disease by suppressing cardiomyocyte apoptosis, inflammation, and oxidative stress, mechanistically linked to targeting SIRT1 (60). In addition, upregulated miR-543 aggravates renal tubular injury in septic acute kidney injury by directly targeting B-cell lymphoma 2 (BCL2), thereby promoting inflammation and apoptosis (61). For those reasons, we supposed that miR-543 may participate in the inflammatory reaction of HCM, which may make a synergic reaction with SNHG1 and MEG8 to regulate the expression of CD163.

We confirmed other regulatory networks, involving ZFAS1/miR-2110/FCER1G and SNHG14/miR-5001-5p/FCER1G axis. FCER1G encodes the Fc receptor *γ* chain, an integral subunit shared by several Fc receptors. This subunit is broadly distributed across diverse immune cell types and orchestrates key immune responses, including phagocytosis and cytokine release (62). Deficiency of FCER1G in non-B cells leads to enhanced and sustained autoreactive B cell responses, an effect mediated through dysregulated CD40L-dependent T cell help, revealing its novel role as a negative regulator of humoral immunity (63). Moreover, FCER1G acts as a critical regulator within the natural killer (NK) cell–CD8^+^ T-cell axis during chronic viral infection, where its absence enhances CD8^+^ T-cell responses and accelerates viral control (64). While the involvement of FCER1G in cardiovascular disease is rarely documented, it is plausible that decreased FCER1G expression exacerbates inflammation in the pathogenesis of HCM.

Beyond its established oncogenic roles in cancers such as hepatocellular carcinoma (65), pancreatic cancer (66), and colorectal cancer (67), SNHG14 has recently been implicated in cardiac pathology. Research indicates that SP1-induced SNHG14 aggravates cardiac hypertrophy by sponging miR-322-5p and miR-384-5p to upregulate protocadherin 17 (PCDH17), making SNHG14 a potential biomarker for cardiac hypertrophy (68). Studies found that lncRNA ZFAS1 expression was increased after myocardial ischemia-reperfusion injury (IRI) (69), while specific overexpression of ZFAS1 can promote cardiomyocyte apoptosis via the mitochondrial pathway (70), which may be the reason for IRI-induced cardiac injury. Further enhancing its clinical relevance, Chang et al. found that a diagnostic panel incorporating ZFAS1 and miR-590-3p with brain natriuretic peptide (BNP) outperformed BNP alone in chronic HF (71). However, the role of lncRNA ZFAS1 in HCM has rarely been studied, which is worthy of further research. As regards miR-2110 and miR-5001-5p, they have not yet been reported. It is very fascinating to investigate whether ZFAS1/miR-2110/FCER1G and SNHG14/miR-5001-5p/FCER1G axis are involved in HCM in further studies.

In this study, we also confirmed that CYP2J2 expression was regulated via the TTN-AS1/miR-6740-3p/CYP2J2 axis. CYP2J2, a member of the monooxygenase cytochrome P450 family, is dominantly expressed in the myocardium, endothelium, and kidneys. CYP2J2 could metabolize arachidonic acid to four epoxyeicosatrienoic acids (EETs) (EETs: 5,6-, 8,9-, 11,12-, and 14,15-EET), which exerted diverse biological effects in cardiovascular diseases (72). Accumulating evidence suggests that both CYP2J2 and its EET metabolites may confer protection against the development of cardiac hypertrophy (73–75). CYP2J2-derived EETs elicit antihypertrophic effects by activating 5′-AMP-activated protein kinase (AMPK) α2, which interacts with and promotes the nuclear translocation of phosphorylated Akt1, thereby enhancing protective transcriptional programs and attenuating pathological cardiac remodeling (73). Another study also indicated that CYP2J2 and its EET metabolites have protected against pathological cardiac remodeling through activation of peroxisome proliferator-activated receptor (PPAR)-γ, resulting in reduced oxidative stress and suppression of NF-κB-mediated inflammation (74). Yang et al. also reported that CYP2J2/EETs confer protection against cardiac fibrosis and dysfunction by blocking pro-inflammatory signal transmission from cardiomyocytes to macrophages (76). To sum up, CYP2J2 protects against cardiac hypertrophy and fibrosis, rendering it a highly promising hub gene for further investigation and a potential therapeutic target in HCM. TTN-AS1 is primarily known for its tumor-promoting functions across multiple cancer types, including esophageal squamous cell carcinoma, breast cancer, and lung cancer (77–79). Lin et al. reported that lncRNA TTN-AS1 was highly expressed in esophageal squamous cell carcinoma (ESCC) tissues and could promote ESCC proliferation and metastasis through the TTN-AS1/miR-133b/FSCN1 regulator*y* axis (77). Another study also indicated that TTN-AS1 enhanced tumor invasion and migration by targeting the miR-4677-3p/ZEB1 axis in lung adenocarcinoma (78). In addition, TTN-AS1 was proven to attenuate sepsis-induced myocardial injury via modulation of the miR-29a/E2F2 axis (80). However, it is limited to knowing the potential role of TTN-AS1 in HCM, which requires further study. For the miR-6740-3p, there were very few reports, as far as we know. Up until recently, Lu et al. reported that miR-6740-3p was found to be up-regulated in Alzheimer's disease (81). Similarly, it is worth exploring whether the TTN-AS1/miR-6740-3p/CYP2J2 axis participates in HCM in the future.

## 5 Limitations

Our study had certain limitations. First of all, HCM-related datasets analyzed in our study were produced utilizing several different platforms, whose batch effect could not be eliminated, and might result in bias. Second, the accuracy of our analysis results may also be affected by our relatively small sample size. Third, although the three hub genes were significantly correlated with immune cells, the precise molecular mechanisms underlying these interactions remain to be elucidated. Finally, while we have employed stringent algorithms and cross-dataset validation to enhance generalizability and robustness, these findings remain hypothetical and necessitate direct validation in an external patient cohort and experimental evidence for the predicted molecular mechanisms to establish definitive biological function.

## 6 Conclusion

In our bioinformatics research, we investigated the hub genes of HCM and constructed a ceRNA network, consisting of SNHG1/MEG8/miR-543/CD163 axis, ZFAS1/ miR-2110/ FCER1G axis, SNHG14/ miR-5001-5p/ FCER1G, and TTN-AS1/miR-6740-3p/CYP2J2 axis. Subsequent immune infiltration profiling revealed significant correlations between these hub genes and distinct immune cell subsets. Collectively, these findings advance the mechanistic understanding of HCM, establish a foundation for subsequent investigation, and highlight potential therapeutic targets.

## Data Availability

The original contributions presented in the study are included in the article/Supplementary Material, further inquiries can be directed to the corresponding authors.

## Footnotes

^1^NCBI GEO: http://www.ncbi.nlm.nih.gov/geo

^2^R: https://www.r-project.org/

^3^BioMart: http://asia.ensembl.org/biomart/martview/

^4^MsigDB: http://software.broadinstitute.org/gsea/msigdb

^5^STRING: https://cn.string-db.org

^6^Cytoscape: http://www.cytoscape.org/

^7^miRDB: http://www.mirdb.org/

^8^miRWalk: http://mirwalk.umm.uni-heidelberg.de

^9^DIANA-LncBase v2: http://www.microrna.gr/LncBase

^10^lncRNASNP2: http://bioinfo.life./lncRNASNP2
