# Supplementary material for: Construction of a competitive endogenous RNA network and identification of potential regulatory axes in hypertrophic cardiomyopathy
Source: Front Cardiovasc Med. 2026 Jan 12;12:1552060. doi: 10.3389/fcvm.2025.1552060 (PMC12832793; doi:10.3389/fcvm.2025.1552060)
Supplement: Supplementary file 1 [file Supplementaryfile1.docx]

**SUPPLEMENTAL MATERIAL**

**Construction of a competitive endogenous RNA network and identification of potential regulatory axes in hypertrophic cardiomyopathy**

**Author:** Rui Gao, MM^1,2,3†^, Meilin Liu, MD^1,2,3†^, Haoyi Yang, MD, PhD^1,2,3^, Lingfeng Zha, PhD^1,2,3^, Ni Xia, MD, PhD^1,2,3^*

^1^ Department of Cardiology, Union Hospital, Tongji Medical College, Huazhong University of Science and Technology.

^2^ Hubei Key Laboratory of Biological Targeted Therapy, Union Hospital, Tongji Medical College, Huazhong University of Science and Technology.

^3^ Hubei Provincial Engineering Research Center of Immunological Diagnosis and Therapy for Cardiovascular Diseases, Union Hospital, Tongji Medical College, Huazhong University of Science and Technology, Wuhan 430022, China.

^†^ These authors contributed equally to this work.

***Address for correspondence**

Ni Xia, MD, PhD, Department of Cardiology, Union Hospital, Tongji Medical College, Huazhong University of Science and Technology; Hubei Key Laboratory of Biological Targeted Therapy, Union Hospital, Tongji Medical College, Huazhong University of Science and Technology; Hubei Provincial Engineering Research Center of Immunological Diagnosis and Therapy for Cardiovascular Diseases, Union Hospital, Tongji Medical College, Huazhong University of Science and Technology, 1277 Jiefang Avenue, Wuhan 430022, China. Telephone: +86-27-85726011, E-mail: nixiaunion@163.com.

**Supplemental Figures and Figure Legends**

**Supplementary FIGURE 1** The top 10 significant terms of GO CCs and MFs enrichment analysis in GSE36961. GO, Gene Ontology; CC, cellular components; MF, molecular functions.
